# Polarization-independent actively tunable colour generation on imprinted plasmonic surfaces

**DOI:** 10.1038/ncomms8337

**Published:** 2015-06-11

**Authors:** Daniel Franklin, Yuan Chen, Abraham Vazquez-Guardado, Sushrut Modak, Javaneh Boroumand, Daming Xu, Shin-Tson Wu, Debashis Chanda

**Affiliations:** 1 Department of Physics, University of Central Florida, 4111 Libra Drive, Physical Sciences Building 430, Orlando, Florida 32816, USA; 2 NanoScience Technology Center, University of Central Florida, 12424 Research Parkway Suite 400, Orlando, Florida 32826, USA; 3 CREOL, The College of Optics and Photonics, University of Central Florida, 4304 Scorpius Street, Orlando, Florida 32816, USA

## Abstract

Structural colour arising from nanostructured metallic surfaces offers many benefits compared to conventional pigmentation based display technologies, such as increased resolution and scalability of their optical response with structure dimensions. However, once these structures are fabricated their optical characteristics remain static, limiting their potential application. Here, by using a specially designed nanostructured plasmonic surface in conjunction with high birefringence liquid crystals, we demonstrate a tunable polarization-independent reflective surface where the colour of the surface is changed as a function of applied voltage. A large range of colour tunability is achieved over previous reports by utilizing an engineered surface which allows full liquid crystal reorientation while maximizing the overlap between plasmonic fields and liquid crystal. In combination with imprinted structures of varying periods, a full range of colours spanning the entire visible spectrum is achieved, paving the way towards dynamic pixels for reflective displays.

## Abstract (web summary)

Plasmonic nanostructures are a promising alternative to conventional pixels, where their characteristics at the nanoscale offer many benefits. Franklin *et al*. combine plasmonic surfaces with liquid crystals to create voltage-tunable polarization-independent color pixels for reflective displays.

## Graphical abstract

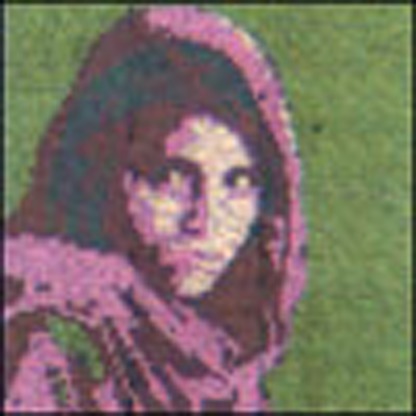

The field of plasmonics has grown over the years due to its unique ability to confine light to subwavelength regions of space. This enhanced confinement has enabled fundamental research on light–matter interactions and, with recent advances in nanofabrication techniques, increased the practical use of plasmonic nanostructures. Many optical applications for these nanostructures have been demonstrated, such as high resolution colour filters^1^,^2^,^3^,^4^, polarizers^5^, broad band absorbers^6^ and selective reflectors^7^,^8^,^9^,^10^,^11^. A key aspect of these devices is the scalability of optical responses with structural dimensions. However, once the respective device is fabricated with a given set of dimensions its optical characteristics remain static^9^,^10^, severely restricting its potential applications. Due to this limitation, much effort has been given into making these plasmonic structures dynamic. One technique is to utilize the anisotropy and reconfigurability of liquid crystals (LCs) to change the dielectric constant surrounding the metallic nanostructure, thereby shifting the plasmon resonance spectral location^12^,^14^. To date, many of these examples deal with infrared or terahertz frequencies^15^,^16^,^17^, and those that are in the visible regime remain limited to a small range of colour tunability due to the modest shifts (∼10–40 nm) in plasmon resonance^18^,^19^,^20^,^21^. While these works show that the phenomenon exists and can be controlled in a variety of ways, they all fall short of the tuning range needed for practical devices.

In this work, we report an increase in this tuning ability up to 95 nm through the use of a highly birefringent LC and a periodic array of shallow nanowells that allow complete LC reorientation and maximum overlap between the plasmonic fields and LC. We develop design rules for maximizing the continuous tuning of plasmonic resonances based on finite-element method (FEM) and finite-difference time-domain (FDTD) simulations, which facilitate accurate predictions of the complex LC orientation on the nanostructured surface and subsequent optical responses. We use the resulting design rules to fabricate a polarization-independent reflective surface where the colour of the nanostructured surface is changed as a function of applied voltage. In combination with nanoimprinted plasmonic surfaces of varying periods, a full range of dynamically tunable colour across the entire visible spectrum is achieved for the first time. The use of LC enables fast millisecond-scale switching times, outperforming present electroactive polymer^22^ and electric/magnetic ink-based^23^ reflective tunable colour technologies. Lastly, to further emphasize the display potential of the system, the resultant colour palette is exploited to form dynamic colour-tunable images. Such an approach can not only lead to large area, thin-film display elements on rigid and flexible substrates, but can also improve the active tunability of general plasmonic and metamaterial systems.

## Results

### LC-plasmonic surface

**Figure 1:**
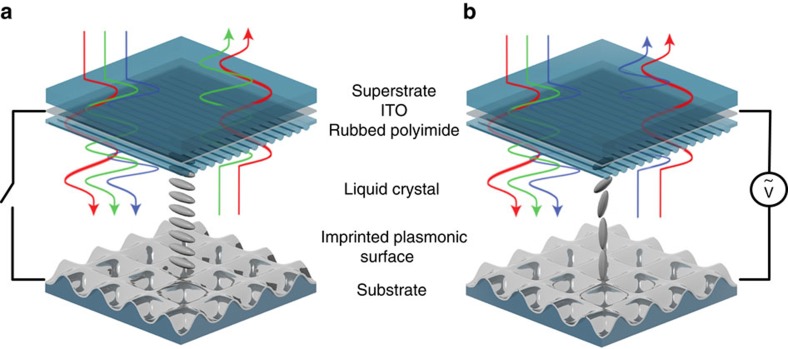
Liquid crystal tunable plasmonic surface. (**a**) Schematic of the plasmonic-liquid crystal cell with impinging white light. Light transmits through the superstrate and liquid crystal layers to interact with the reflective plasmonic surface. The surface selectively absorbs light while reflecting the rest back out of the device. The wavelength of this absorption depends on the liquid crystal orientation near the interface which in turn depends on the electric field applied across the cell. (**b**) An applied electric field across the cell reorients the liquid crystal and changes the wavelengths of absorbed light.

Figure 1 shows an illustration of the LC-plasmonic coupled system. The surface consists of a shallow imprinted array of nanowells coated with a continuous layer of aluminium. The surface is used as part of a LC cell, in which the nanostructured aluminium serves as a bottom electrode. The other half of the LC cell consists of an indium tin oxide (ITO) coated glass substrate, a polyimide alignment layer rubbed diagonally with respect to the grating vector of the plasmonic nanostructure, and chopped silica spacers. A high birefringence (Hi-Bi) LC is placed inside the cell in direct contact with the aluminium surface. Unpolarized white light transmits through the top glass and LC layers and couples to the plasmonic modes of the aluminium surface. The spectral location of these surface plasmon resonances (SPR) is dependent on the surrounding dielectric constant and is determined by the LC's orientation, which in turn, is controlled through the applied electric field between the ITO layer and aluminium surface. Light which is not absorbed by the surface is reflected back out of the LC cell to be perceived as a visible colour. Figure 1a,b illustrates a single surface as the LC reorients between its two extrema and the resulting colour change. This method differs greatly from standard LC displays in which colour is generated by static polymeric filters and the LC, in conjunction with polarization layers, functions as a light valve.

To achieve a large plasmonic shift, several design considerations are taken into account. A positive dielectric anisotropy nematic LC is used which, in general, has a larger dipole moment than its negative counterpart. This lowers the electric field required to reorient the LC while also increasing its birefringence. This is important as the shift in plasmonic resonance is proportional to the LC's birefringence. We therefore use a commercially available Hi-Bi LC (LCM1107, LC Matter Corp.) with *n*_e_=1.97 and *n*_o_=1.55, and a resultant birefringence of 0.42. Second, the vertical electrode configuration allows for the plasmonic surface to serve as an electrode. This also increases the ability of an applied field to reorient the LC near the aluminium surface as compared with the fringe fields generated by an in-plane-switching device. With this electrode configuration and LC polarity, the OFF-state LC orientation must be parallel (homogeneous) to the plasmonic surface to allow reorientation with an applied field. This places several constraints on the nanostructure dimensions and constituent materials. It has been shown that the orientation of LC on a nanostructured surface is highly dependent on the space in which it is confined^24^,^25^,^26^, that is, if the well depth-to-diameter ratio is too large, the LC aligns vertically (homeotropic alignment) inside the well^27^. For this reason, the nanowells must be shallow to allow the homogeneous alignment of the LC inside. The orientation of the LC near the surface is also material dependent. Various low loss plasmonic metals, such as silver, exhibit homeotropic alignment due to their surface energy^28^, ultimately inhibiting tunability. While gold has been used extensively in LC tunable plasmonics, its intrinsic intraband absorption in the visible spectral domain makes it unsuitable for full visible colour generation. For these reasons, aluminium is preferred as the tunable plasmonic surface as it has been shown to impart degenerate planar alignment^29^ without intrinsic visible domain absorption. Degenerate anchoring implies that the LCs do not have a preferred alignment direction within the plane of the surface.

### LC orientation on nanostructured surfaces

**Figure 2:**
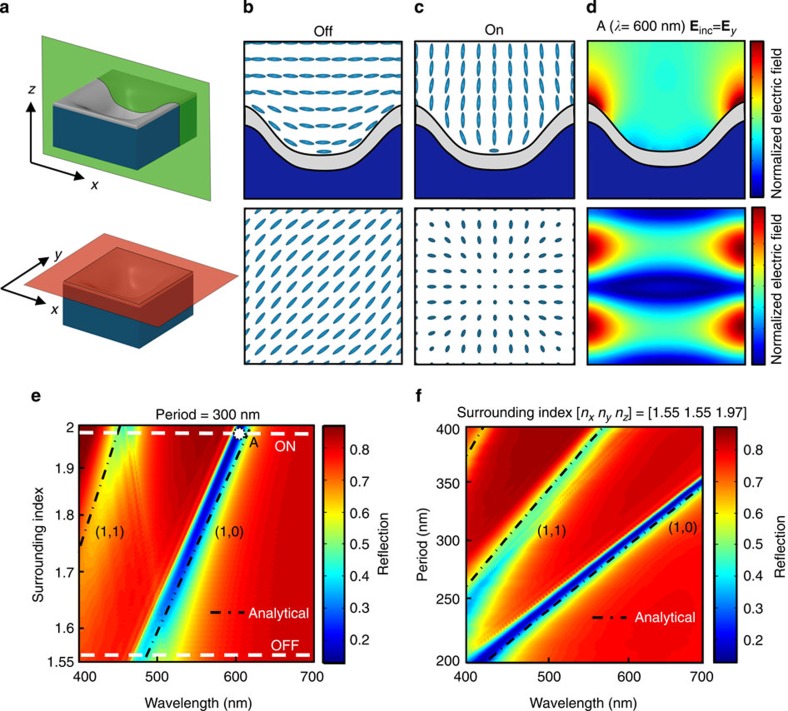
Liquid crystal orientation states and plasmonic modes. (**a**) Schematic top and cross-sectional views of the nanostructure unit cell. The green and orange planes represent *x*–*z* and *x*–*y* cross-sections, respectively. (**b**,**c**) FEM-computed liquid crystal orientation on a 300 nm period nanostructure (**b**) without an applied electric field (OFF) and (**c**) with a field of 10 V μm^−1^ (ON). (**d**) FDTD-computed electric field intensity (|**E**|^2^) spatial cross-section of the first order plasmonic resonance at *λ*=600 nm, showing penetration of the fields into the liquid crystal region. The 300 nm period structure is excited with *y*-polarized light. (**e**) FDTD-predicted reflectance spectrum as a function of surrounding index for a structure of period 300 nm. White dashed lines indicate the effective surrounding index for the OFF and ON states, respectively. Black dashed lines show the analytical dispersion relation for grating-coupled surface plasmon modes. (**f**) FDTD predicted reflectance spectrum as a function of structure periodicity for the anisotropic effective index given by the ON liquid crystal orientation state, [*n*_*x*_ *n*_*y*_ *n*_*z*_]=[1.55 1.55 1.97]. Black dashed lines show excellent agreement with the analytical dispersion relation.

The LC orientation within and near the nanostructured surface is vital in defining the spectral location of the plasmonic modes and ultimately their potential for being tuned. To understand the structure's topographical influence on the LC, FEM calculations on a unit cell of the surface is performed. The numerical simulation uses a Q-tensor method to minimize the Landau-de Gennes-free energy functional for a given set of boundary conditions, LC parameters and external applied fields^30^. The LC will take the orientation, which minimizes this internal energy, the unit cell and results of which can be seen in Fig. 2a–c. The LC depicted in Fig. 2b–c represents the average local LC orientation about a uniformly sampled grid. The LC is not drawn to scale as typical molecules are ∼2-nm long while the structure period is 300 nm. The simulations use periodic boundary conditions to imitate an infinite array of nanowells and use experimentally verified LC elastic coefficients (see Supplementary Fig. 1). Degenerate anchoring is applied for the aluminium surface, while the top surface anchoring energy is set to zero. The purpose of this is to isolate the aluminium surface's alignment properties from that of the top polyimide alignment layer. Without an external bias, the LC conforms to the profile of the aluminium surface and aligns diagonally with respect to the unit cell as can be seen in the FEM prediction of Fig. 2b. With the application of voltage, a Freedericksz transition is observed where the LC molecules start to reorient from their initial OFF state. Further increase in voltage continuously rotates the LCs vertically until they align along the electric field as shown in FEM prediction, Fig. 2c. This transition followed by a continuous tuning can be seen in Supplementary Fig. 2, where the experimental reflection spectra of a structured surface is tuned as a function of voltage. These orientation matrices along with the *n*_e_ and *n*_o_ values of the LC produce an anisotropic index tensor, which can be used to predict properties of the surface's optical behaviour. Interestingly, the orientation states, and therefore index tensors, have symmetries which suggest polarization-independent behaviour from light polarized along the structure's orthogonal periodicity vectors. This is a useful property for reflective display elements illuminated with ambient white light as polarizers are not needed, reducing fabrication costs and increasing reflection efficiency. Lastly, it's important to note that the actual LC orientation within a device will also depend on the top alignment layer and the spacing between them. To maintain the system's polarization independence, the LC orientations in Fig. 2b,c must be preserved. For the present case, a relatively large cell gap of ∼4 μm (defined by the order coherence length of the specific LC) is used to reduce the effect of the top-rubbed-polyimide alignment layer on the anchoring of the aluminium surface. Polarization-dependent reflection is observed for cell gaps at ≤2 μm due to the strong influence of the top alignment layer. Cell gap measurements are obtained by fitting FTIR reflection spectra to a Febry–Perot analytical model (see Supplementary Fig. 3).

### Plasmonic mode dispersion

Once the LC orientation states are found, their effect on the plasmonic surface can be determined. Figure 2d shows the FDTD-predicted cross-sectional and top view electric field distributions for the first order resonant wavelength at 600 nm. The structure is excited from above at normal incidence with *y*-polarized light and has a period of 300 nm, a 100-nm nanowell depth and a 30-nm thick aluminium layer. The evanescent plasmonic fields penetrate tens of nanometers into the surrounding material and define the region sensitive to LC reorientation^12^. The refractive index component normal to the metallic surface within these regions changes dramatically between the two LC orientation states, due to near 90° LC rotation, allowing the maximum theoretical shift in plasmon resonance for the given LC and nanostructured plasmonic surface. Furthermore, the continuous metallic surface forces these fields into the LC region, maximizing the overlap between plasmonic fields and LC. This results in an increased tuning capability compared with discontinuous plasmonic systems, where fields can be partially confined in untunable dielectric regions inaccessible to the LC. As discussed above, regions most sensitive to LC reorientation are those with high plasmonic fields.

To elucidate the plasmonic modes of the surface, we average the LC orientations in Fig. 2b,c within the plasmonic fields (1/e) of Fig. 2d to obtain anisotropic effective index values for the OFF ([*n*_*x*_ *n*_*y*_ *n*_*z*_]=[1.72 1.72 1.56]) and ON ([*n*_*x*_ *n*_*y*_ *n*_*z*_]=[1.55 1.55 1.97]) device states. Figure 2e shows the reflection spectrum for normal angle of incidence as a function of the effective refractive index taken between these anisotropic index states. A linear shift in SPR is observed with the increase in the effective refractive index, resulting in a continuous variation of colour. The dashed black lines indicate grating-coupled propagating surface plasmon (GCSP) modes defined by the analytical equation, 
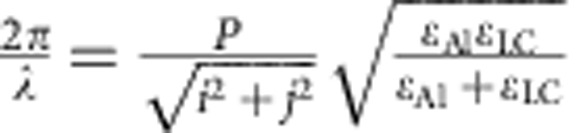
, where *P* is the period of the grating, *i* and *j* are mode orders, and *ɛ*_Al_ and *ɛ*_LC_ are the permittivity for aluminium^31^ and surrounding LC, respectively. This dispersion relation sets a limit on the maximum active shift obtainable for a given LC-GCSP system and is proportional to the LC's birefringence, *n*_e_−*n*_o_. The Hi-Bi LC used herein has a *n*_e_ and *n*_o_ of 1.97 and 1.55, respectively, giving a maximum first order resonance shift of 110 nm from the GCSP analytical expression. This assumes complete LC reorientation and overlap between the LC index change and plasmonic mode profile. Dashed white lines in Fig. 2e indicate the two effective index extrema predicted by the LC orientation states in Fig. 2b,c. As per the grating-coupled SPR equation mentioned above, similar tuning of SPR can be accomplished by changing the period of the nanostructure. Figure 2f shows the FDTD-predicted far-field reflection from the surface as a function of period for the ON state surrounding anisotropic index of [*n*_*x*_ *n*_*y*_ *n*_*z*_]=[1.55 1.55 1.97]. Dashed black lines show excellent agreement between FDTD predictions and the analytical GCSP dispersion relation in both Fig. 2e,f. Diffraction is a concern when using periodic structures. For the grating period of 300–380 nm, diffraction occurs below 460–590 nm wavelength range but with low efficiency due to the shallowness of the nanostructure. Furthermore, the weak diffracted light below this cutoff wavelength range diffracts at angles greater than the total internal reflection angle of the top glass–air interface, effectively trapping them within the LC cell.

### Colour-tunable reflective surfaces

**Figure 3:**
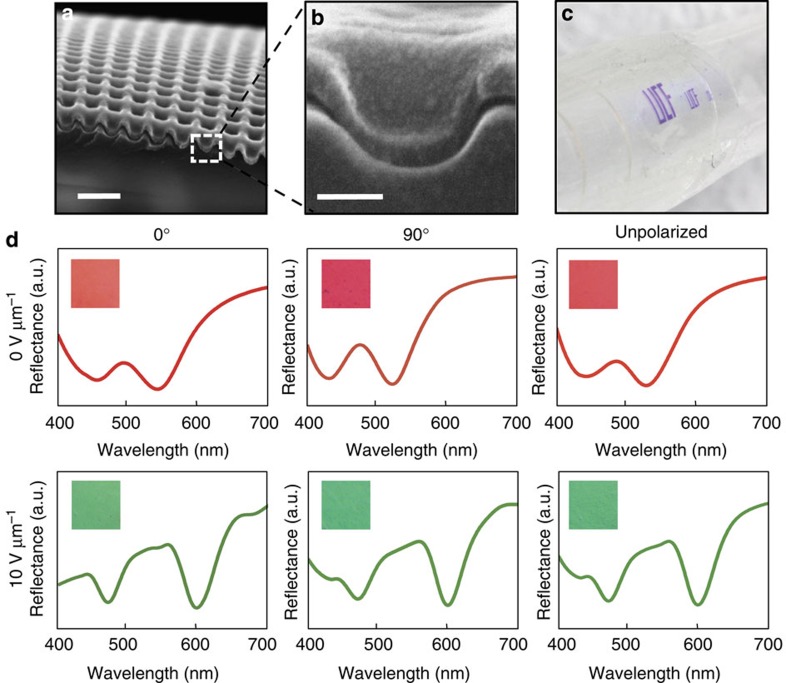
Fabricated structure and polarization analysis. (**a**) SEM image of the structure with period 300 nm before fabrication into liquid crystal cell and (**b**) a close up of the nanowell unit cell. Scale bars, (**a**) 500 nm, (**b**) 100 nm. (**c**) Optical image of macroscopically patterned ‘UCF' on a flexible PET substrate. (**d**) Reflection spectra of a 320-nm period metasurface for various polarization and voltage states. The polarization states are defined as the angle between the *x*-direction grating vector and the optical axis of the polarizer. Insets are microscope images depicting the reflected colour. Line colours are determined by the CIE colour-matching functions for the respective spectra.

Figure 3a,b shows a scanning electron microscope (SEM) image of the structured aluminium surface before LC cell assembly. A simple nanoimprinting technique is employed to pattern a polymer film (SU-8) followed by a blanket deposition of ∼30 nm aluminium using an electron beam evaporator. The master patterns are fabricated through direct laser writing (DLW). One such DLW master can produce hundreds of polymeric imprinting stamps, and one such stamp can produce thousands of imprints without any noticeable pattern degradation. The process is compatible with rigid as well as flexible substrates as can be seen in Fig. 3c, where a macroscopically patterned ‘UCF' LC-plasmonic surface is formed on a conformal plastic (polyethylene terephthalate (PET)) surface.

To determine the polarization dependence of the LC-plasmonic system, microscope images and reflectance spectra are shown in Fig. 3d for a nanostructured surface of 320 nm period. Polarized states are defined by the angle between the *x*-direction grating vector of the surface and the optical axis of the polarizer. Insets are microscope images showing the reflected colour while line colours are determined by the International Commission on Illumination (CIE) colour-matching functions for the respective reflection spectra. While slight variations in spectra are observed, they are too minor to drastically affect the perceived colour. This shows that the system is largely polarization independent, a finding consistent with LC orientation simulations.

**Figure 4:**
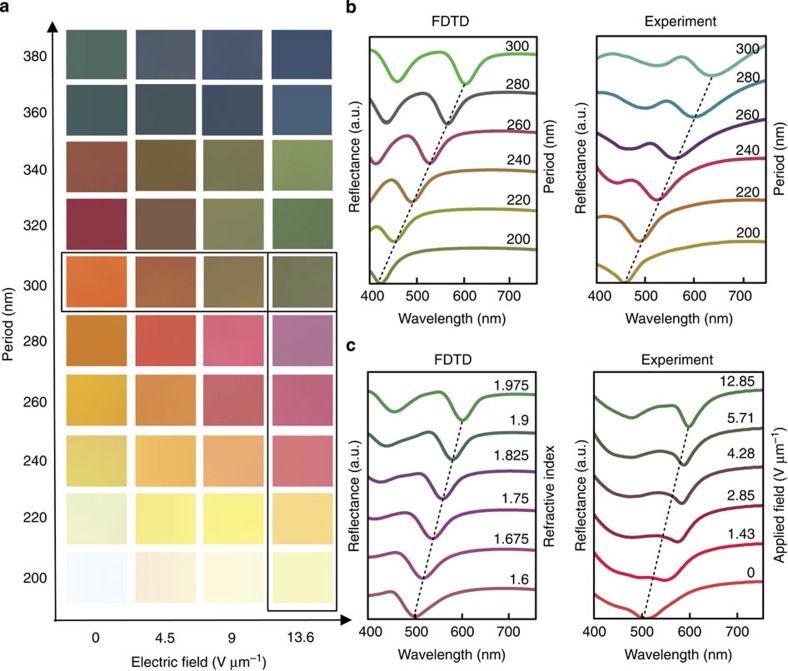
Measured and predicted colour palette. (**a**) The palette of obtainable colour for structures of period between 200 and 380 nm as a function of applied voltage. (**b**) Simulated and experimental reflectance spectra of the selected surfaces as a function of period with the ON state anisotropic effective index [*n*_*x*_ *n*_*y*_ *n*_*z*_]=[1.55 1.55 1.97] and 13.6 V μm^−1^ applied electric field, respectively. Dashed black trend lines show the linear relationship between plasmonic absorption and periodicity. (**c**) Simulated and experimental reflectance spectra of the surface with period 300 nm as a function of surrounding index and applied electric field, respectively.

The full range of colours obtainable with the LC-plasmonic system as a function of nanostructure period and applied electric field can be seen in Fig. 4a. Having the first (1,0) and second (1,1) order grating-coupled SPRs within the optical spectrum allows a full range of colours (that is, red, green and blue (RGB) and cyan, yellow and magenta (CYM)) as compared with single resonance subtractive colour, which is limited to the CYM colour space. This is further supported by the full circle of points about the central white point in the CIE chromaticity diagram depicted in Supplementary Fig. 4. We emphasize that LC on planer aluminium or nanostructured polymer does not generate colour and only with the combination of these two components does colour result (see Supplementary Fig. 5). Figure 4b,c shows experimental and theoretical reflection spectra corresponding to the structural colours outlined in Fig. 4a. Due to a ∼±20 nm deviation in structural periodicity during DLW, the FDTD reflection spectra of Fig. 4b,c are guassian-weighted averages about the indicated period. We use guassian-weighted averaging as larger deviations from the ideal period are less probable. The CIE colour-matching functions are used to obtain the line colour for each plotted spectra.

A close linear relationship between structure period and plasmonic absorption location is observed in Fig. 4b and shown by the dotted line. Figure 4c shows the FDTD-predicted and experimentally measured reflection spectrum from a single structure of period 300 nm as a function of anisotropic effective surrounding index and applied voltage, respectively. Here we observe a nonlinear relationship between the applied voltage and resonant wavelength. We believe this due to the bulk of the LC within the plasmonic field reorienting with a relatively small applied electric field (0–2 V μm^−1^). However, a much greater voltage is required to reorient the LC near the nanostructure due to the aluminium surface anchoring forces. The first order experimental resonance shifts of 95 nm are reasonably close to the maximum FDTD and analytically predicted value of 110 nm (compare Fig. 2e with Fig. 4c). Slight deviations between theoretical and experimental observations are believed to originate from fabrication tolerances. While the spectra in Fig. 4b,c are offset to emphasize the shift in plasmonic resonance, the absolute reflection (or efficiency) has peaks from 50 to 80% over the visible domain, an example of which can be seen in Supplementary Fig. 2.

**Figure 5:**
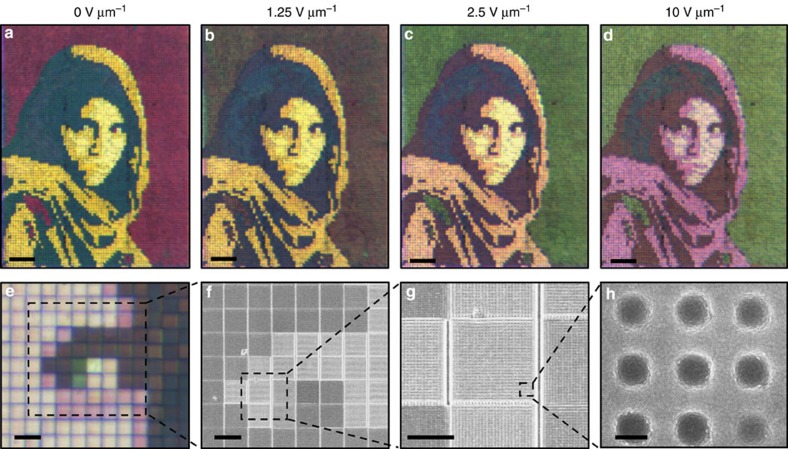
Dynamic colour tuning of arbitrary images. (**a**–**d**) Microscope images of a singular Afghan Girl image as a function of applied electric field. Nanostructure periods are chosen so colours match the original photograph at colour tuning saturation, 10 V μm^−1^. Scale bars (**a**–**d**), 100 μm. Defects due to fabrication errors (missing pixels) have been replaced by nearest neighbours. (**e**) Microscope image at 10 V μm^−1^ with a × 10 objective showing pixilation of the image. (**f**–**h**) SEM images of the sample before fabrication into a liquid crystal cell. The series shows the constituent nanostructure of individual pixels. Scale bars, (**e**) 20 μm, (**f**) 10 μm, (**g**) 5 μm, (**h**) 150 nm. Copyright Steve McCurry/Magnum Photos. Image rights granted by Magnum Photos New York.

To emphasize the display potential of these plasmonic surfaces, the resultant colour palette is exploited to form colour-tunable images. Figure 5a–d shows 0.75 × 1 mm reflective optical images of the Afghan Girl (Magnum Photos), while the singular sample is tuned through 0–10 V μm^−1^ of applied electric field. The colour mapping process is outlined in Supplementary Fig. 6. From the Afghan Girl image, it is evident that the colour of nanostructured surfaces with different periodicities saturate at different voltages. For example, the background saturates to its final colour (green) at 2.5 V μm^−1^, where the shawl saturates (to red/pink) at 10 V μm^−1^. This can be attributed to the surface anchoring force dependence on topography, which varies with the periodicity of the metasurface. Figure 5e shows a magnified image of the Afghan Girl using a × 10 objective. The 10 × 10 μm pixels can be seen in the following SEM subfigures (Fig. 4f–h) as two-dimensional (2D) gratings of varying periods. In this example, the pixelated plasmonic surface is singularly addressed and therefore limited to display one image. Further addressing of each pixel based on a standard addressing scheme will enable the display of video.

We used a × 4 objective with 0.07 numerical aperture (NA) for the Afghan Girl images in Fig. 5a–d and a × 10 objective with 0.25 NA for the image in Fig. 5e. These NAs correspond to collection angles of 4° and 15°, respectively, between which we don't see an appreciable difference in colour. However, the present structure is angle dependent due to the excitation of GCSPs and rigorous coupled wave analysis has been performed to quantify this. Supplementary Fig. 7 shows the polarization-averaged angle-dependent reflection of a 300 nm period structure with a colour bar showing the CIE-predicted colour of the surface for each individual angle. From this we find the colour of the structure relatively angle invariant up to ∼20°.

It should also be noted that the GCSP resonance relies on multiple periods of the nanostructure. This means a minimum pixel size should exist for a given periodicity, below which one would observe a change in reflected colour. Supplementary Fig. 8 shows the reflection spectrum as a function of the number of nanostructure periods. We find that seven periods are required for the resonant wavelength to approach that of the infinitely periodic case. For the 300 nm period structure simulated, this predicts a minimum pixel size of 2.1 μm. As the pixel size decreases below the surface plasmon propagation length, the reflected colour shifts until it begins to wash out due to the weakening of the resonance. Lastly, we note that the grey states are not achievable by the LC-plasmonic surface alone. Even in standard LC displays, the colour-generating mechanism is independent of the grey state mechanism. For example, static polymer filters, which generate colour, are laminated onto a LC cell, which controls the grey state. Similarly, separate layers of polarization optics would be needed to achieve grey states in the present LC-metasurface device.

## Discussion

LCs offer the added benefit of millisecond-scale response times needed for high temporal resolution video. While Hi-Bi nematic LCs tend to have increased viscosities and decreased reorientation times compared with standard nematic LCs, the system in question can complete voltage cycles under 90 ms without any optimization (see Supplementary Fig. 9). Other systems for active structural colour, such as electroactive polymers^22^ and electric/magnetic ink^23^, often require seconds to tens of seconds to change colours, severely limiting their use in displays and other electro-optic devices. Another critical advantage of the tunable plasmonic surface is that the number of subpixels in a display device can be reduced, therefore increasing pixel density and resolution. Instead of three colour-generating subpixels, RGB or CYM of present displays, two dynamic colour pixels can have the same colour-producing abilities. It remains a challenge though to span the entire spectrum using a single pixel. We anticipate further improvements in tunability by exploiting different plasmonic coupling mechanisms, polarization dependence and bistability of LC orientation states.

In summary, we have presented a polarization-independent LC-plasmonic system, which shows continuous colour tuning over a large range of the colour spectrum. Using Hi-Bi LC's with a continuous nanostructured aluminium surface, the grating-coupled SPRs can be tuned over a large range. This range of tunability approaches the maximum theoretically predicted value through an engineered surface, which ensures significant LC reorientation and maximum overlap between the plasmonic fields and LC. By varying the period of the nanostructure, a full range of visible colour is achieved. The presented framework makes LC-plasmonic systems more attractive candidates for display, filter and other actively tunable optical technologies.

## Methods

### Fabrication of plasmonic surfaces

The plasmonic surfaces are fabricated through DLW using a commercially available femtosecond laser lithography system (NanoScribe GmbH). The Dip-In configuration was used with a × 100, 1.3 NA oil immersion objective (Zeiss) and IP-Dip (NanoScribe GmbH) photoresist. The IP-Dip is drop cast on a fused silica substrate and the objective immersed directly in the photoresist. After writing, samples were developed in propylene glycol monomethyl ether acetate (Sigma-Aldrich) for 20 min, rinsed in isopropyl alcohol and dried over a hot plate. The structures are sinusoidal in nature due to the ∼50–100 nm ovular voxel resolution limitations of the DLW system. The patterned polymeric substrates are then either taken to the next step of metallization or used as a master for nanoimprint lithography for rapid replication. If used for nanoimprint lithography, a polymer (dimethylsiloxane) (Dow Corning, Sylgard) mold is cast from the sample. A thin film of SU-8 2000.5 (MicroChem) was spun (500 r.p.m. for 5 s followed by 3,000 r.p.m. for 30 s) then prebaked at 95 °C for 1 min. This film is imprinted with the polymer (dimethylsiloxane) mold and then ultraviolet cured (1 min) and post-exposure baked (95 °C for 1 min). The substrates for the imprinted SU-8 are glass and PET (Sigma-Aldrich) coated with ITO for rigid and flexible samples, respectively. To avoid the glassing temperature of PET, pre- and post-exposure baking temperatures were reduced to 80 °C but baked at doubled times.

### Electron beam deposition

The 30-nm Al films are deposited using a Temescal (FC-1800) six-pocket electron beam evaporation system. For smooth quality films, the sample is mounted on a thermal electric cooler (TEC) and brought to −20 °C. Evaporations are done at pressures of ∼6 × 10^−6^ Torr and deposition rates of ∼0.1 nm s^−1^. Before deposition, three edges of the sample were masked off. This greatly reduces the chance of a short circuit in the completed LC cell.

### LC cell formation

The plamsonic LC cell is fabricated using commercially available twisted nematic LC cells (AWAT PPW, Poland). The commercial cells are heated to 200 °C and then split into two rubbed-polyimide ITO-coated glass slides with 5-μm silica spacers. A single slide is adhered to the plasmonic surface sample using NOA 81 with the polyimide alignment diagonal to the nanostructure grating vector. Once ultraviolet cured, the LC-plasmonic cell is heated to 100 °C and infiltrated with LC (LCM1107). The cell is then allowed to cool down to room temperature. The LC cells are driven with a 1 kHz AC sine wave to reduce ion migration. All reported voltages are root mean square values.

### Optical measurements and images

Reflection spectra are collected using a × 10, 0.07 NA objective on an optical microscope (Hyperion 1000) coupled to a Fourier transform infrared spectrometer (Vertex 80) and outfitted with a 0.6-mm spatial aperture. Reflection spectra are normalized to an aluminium mirror with 96% reflectivity. Images are collected using the same optical microscope with × 4 and × 10 objectives and an Infinity 2–5 camera. Defects due to laser lithography patterning errors (missing pixels) have been replaced by nearest neighbours in Fig. 4a–d with the GIMP software package.

### FEM modelling

The orientation of the LC within and near the nanostructures are numerically simulated using a FEM program created by the LC modelling group at UCL^30^. The program uses a Q-tensor method to minimize the Landau-de Gennes free energy functional for a given structure geometry, LC parameters and external electric field. The method utilizes three elastic constants (bend, splay and twist), fourth-order terms in the bulk free energy and Rapini–Papoular surface potentials. The nanowell surface profile is imported from SEMs and set to have degenerate alignment, while the top rubbed-polyimide alignment layer is homogeneous. LC parameters are taken from company provided, but experimentally verified, product information (see Supplementary Fig. 1). The simulation uses adaptive tetrahedral meshing with a density of 0.5 and 0.07 points per nm^3^ in the bulk LC and near the aluminium surface, respectively.

### FDTD modelling

Reflection spectra are calculated using experimental parameters for the printed 2D grating structures, with commercial FDTD software package (Lumerical FDTD, Lumerical Solutions). The profile for the electromagnetic simulations was obtained by fitting an analytical equation to SEMs of the nanostructured surface (Fig. 3b). Through further simulations, it was confirmed that the form of the equation has a small impact on the reflection spectrum but not resonance location, as *λ*_res_ strictly depends on structure periodicity due to the phase-matching requirement of GCSPs. The wavelength-dependent refractive index of aluminium is taken from ref. 31 and the anisotropic parameters of the LC layer are obtained using an effective index model based on the orientation of LC obtained from FEM calculations. Slight variations in the dimensions of the DLW structures, the properties of the constituent materials and the levels of control (for example, uniformity, surface roughness and so on) associated with the fabrication are the likely causes of the 10–15% overall discrepancy between experimental observation and FDTD simulation.

## Additional information

**How to cite this article:** Franklin, D. *et al*. Polarization-independent actively tunable colour generation on imprinted plasmonic surfaces. *Nat. Commun.* 6:7337 doi: 10.1038/ncomms8337 (2015).

## Supplementary Material

Supplementary Information Supplementary Figures 1-9
