# Supplementary material for: Polarization-independent actively tunable colour generation on imprinted plasmonic surfaces
Source: Nat Commun. 2015 Jun 11;6:7337. doi: 10.1038/ncomms8337 (PMC4490413; doi:10.1038/ncomms8337)
Supplement: Supplementary Information — Supplementary Figures 1-9 [file ncomms8337-s1.pdf]

a

LCM-1107 Elastic, Electro-optic, and Thermal Properties

At 25°C, 1 kHz:

V_{th} [V_{rms}]	1.6
$\varepsilon_{\|\|}$	19.8
$\varepsilon_{\perp}$	4.2
$\Delta\varepsilon$	15.6
K_{11} [pN]	21
K_{33} [pN]	32
K_{33}/K_{11}	1.5

At 25°C, 633 nm:

Δn	0.38
g_1/K_{11} [ms mm^{-2}]	10.5
FoM [mm^2 s^{-1}]	13.8

Phase Transition Temperatures:

Crystal-Nematic	-10°C
Nematic-Isotropic	100°C

b

LCM-1107 Dispersion at 25°C

λ [nm]	n_e	n_o	Δn
450	2.004	1.5586	0.4417
486	1.9723	1.5516	0.4207
546	1.9414	1.5429	0.3985
589	1.9266	1.5382	0.3884
633	1.9150	1.5350	0.3800
656	1.9108	1.5328	0.3780

Supplementary Figure 1 | Liquid crystal properties. (**a**) Elastic, Electro-Optic and Thermal properties of LCM1107. (**b**) Dispersion of LCM1107. These properties are used in FEM and FDTD simulations.

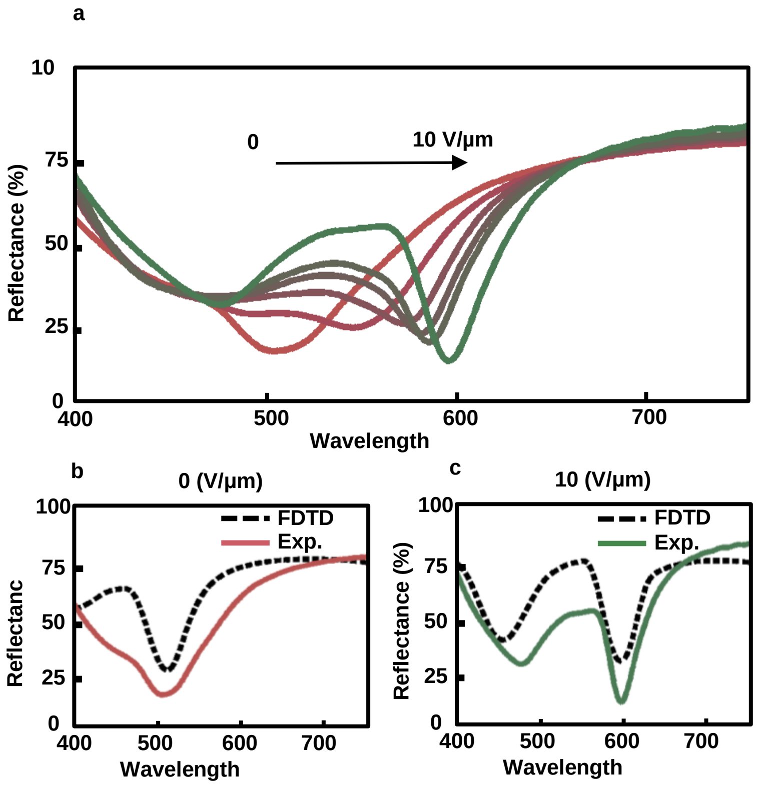

Supplementary Figure 2 | Absolute reflection from the LC-metasurface system. (**a**) FTIR reflection spectra of a period 300 nm metasurface as a function of voltage. Line color is found from the CIE color matching functions. Fabry-Perot oscillations have been filtered to more easily convey plasmonic absorption. This shows the continuous nature of the LC-plasmonic reversible tuning. (**b-c**) compares the FDTD predicted response to experiment for the 0 V μm^{-1} and 10 V μm^{-1} cases, respectively. These figures show the absolute reflection (or efficiency) of the LC-plasmonic system.

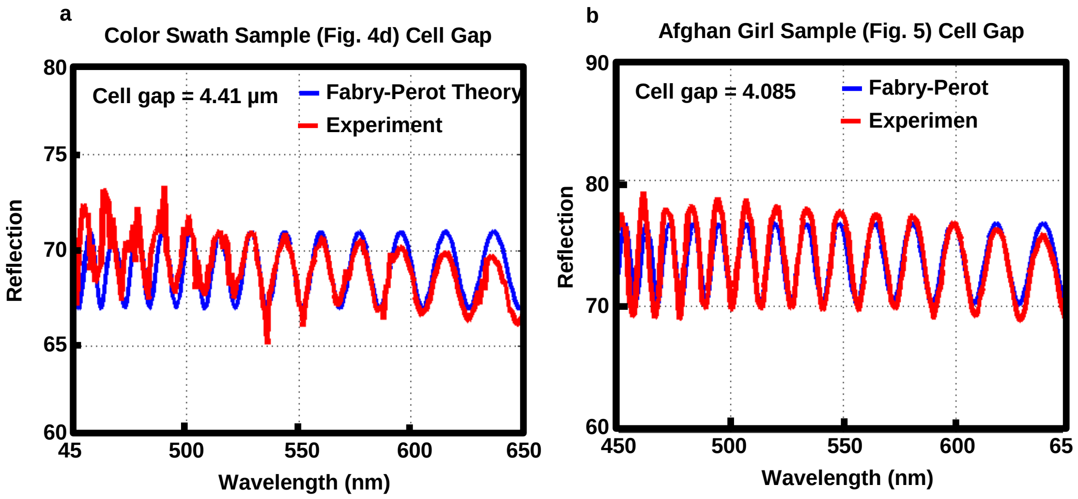

Supplementary Figure 3 | Cell gap measurements. (**a-b**) FTIR reflection measurements off-pattern with matching Fabry-Perot theory to determine cell gaps for devices used in Figs 4 and 5, respectively. 15 V μm^{-1} is applied across the cells to guarantee a vertical LC orientation state.

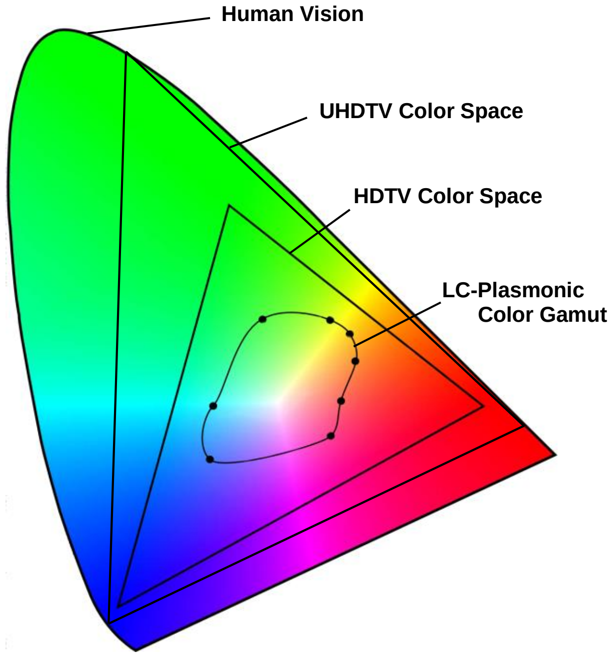

Supplementary Figure 4 | CIE Chromaticity Diagram. (**a**) Dotted line obtained from the outermost colors of the color swath of Fig. 4a, mapped with the color matching functions to the CIE chromaticity diagram. The HDTV color space is defined by the ITU-R BT.709 standard, while UHDTV color space is defined by the ITU-R BT.2020 standard. While absorptive in nature and therefore low in color contrast, the LC-plasmonic system can create a full range of colors about the central white point of the diagram.

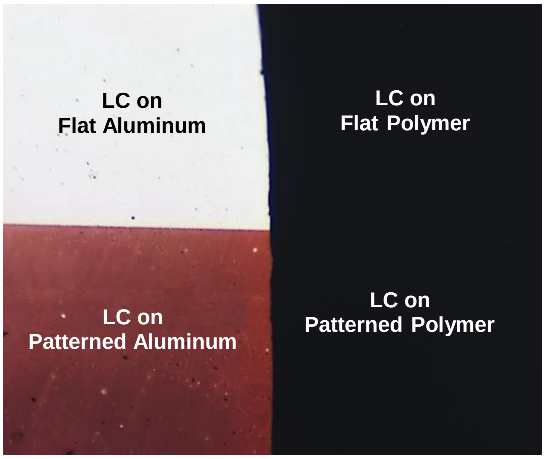

Supplementary Figure 5 | Color Origin. To reinforce the origin of the reflected color, the above shows an optical micrograph containing four distinct regions, all of which contain LC. The first, showing flat aluminum, reflects all light. The second contains flat polymer without aluminum and is dark due to negligible reflection. The third consists of patterned polymer without aluminum, which is also dark. The fourth, containing nanostructured aluminum, reflects a given color. Together, these regions show that structure, aluminum, and LC are not individually responsible for color generation, and only through the combination of all three, does color result.

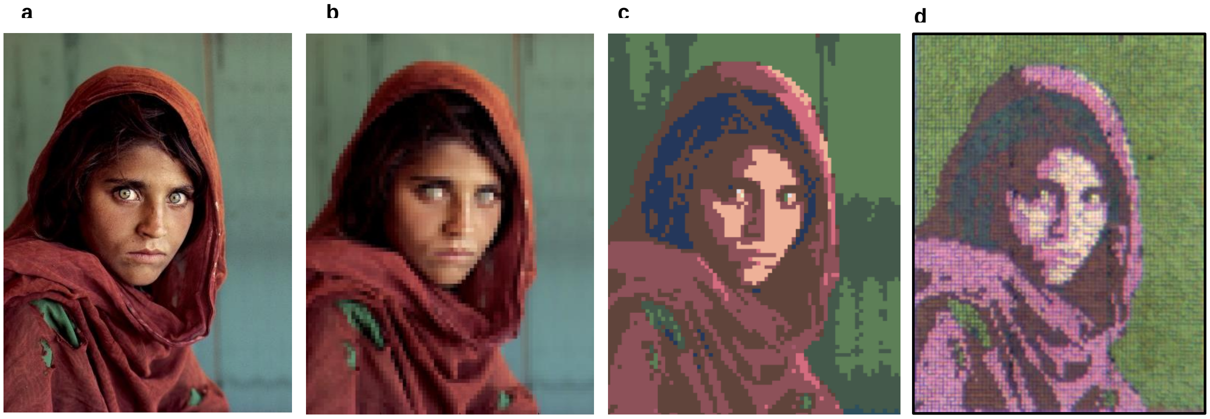

Supplementary Figure 6 | Color mapping to arbitrary images. (**a**) An arbitrary image is (**b**), pixilated to a desired dimension. In this case the image used is the Afghan Girl (Copyright Steve McCurry / Magnum Photos. Image rights granted by Magnum Photos New York). A database is made of the color swath in Fig. 4a, mapping Lab color space to direct laser writing (DLW) parameters. (**c**) The pixilated image in (**b**) is mapped using a Lab space least distance method to the DLW settings from the database. (**d**) Optical micrograph of the ON state (10 V μm^{-1}) display.

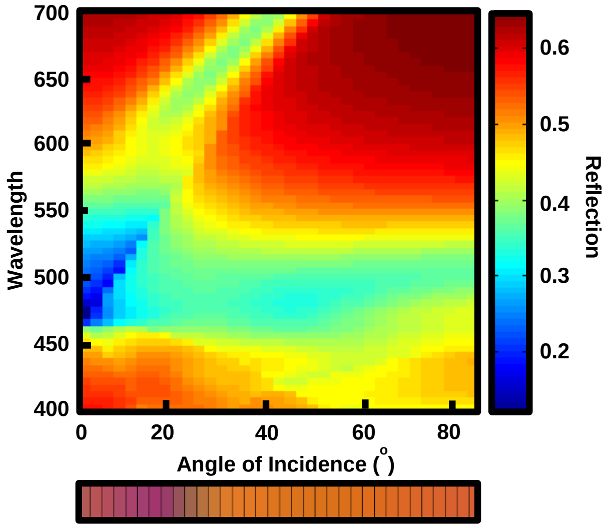

Supplementary Figure 7 | Angle Dependence. Rigorous coupled wave analysis (RCWA) simulation for the reflection of a 300 nm period metasurface as a function of incident angle. Structure is excited with unpolarized white light. We find the color of the structure is invariant up to ~20°, upon which we see a change due to the splitting of the plasmonic mode.

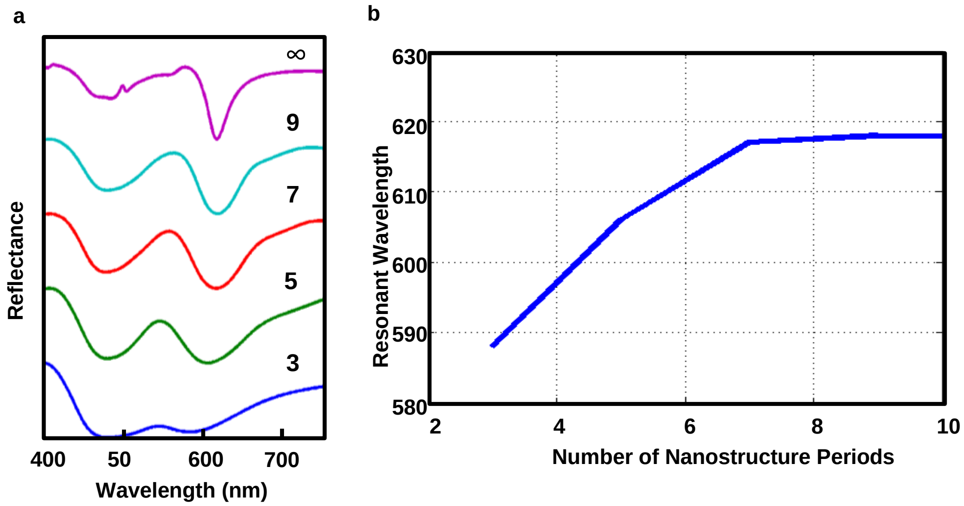

Supplementary Figure 8 | Pixel size dependence. FDTD simulations for a structure of period 300 nm and 100 nm relief depth. (**a**), FDTD reflection spectra of structures with varying numbers of periods. (**b**) The first order resonant wavelength as a function of surfaces with varying numbers of periods. We find that the resonant wavelength location approaches that of the infinitely periodic structure within 1 nm for 9 structure periods.

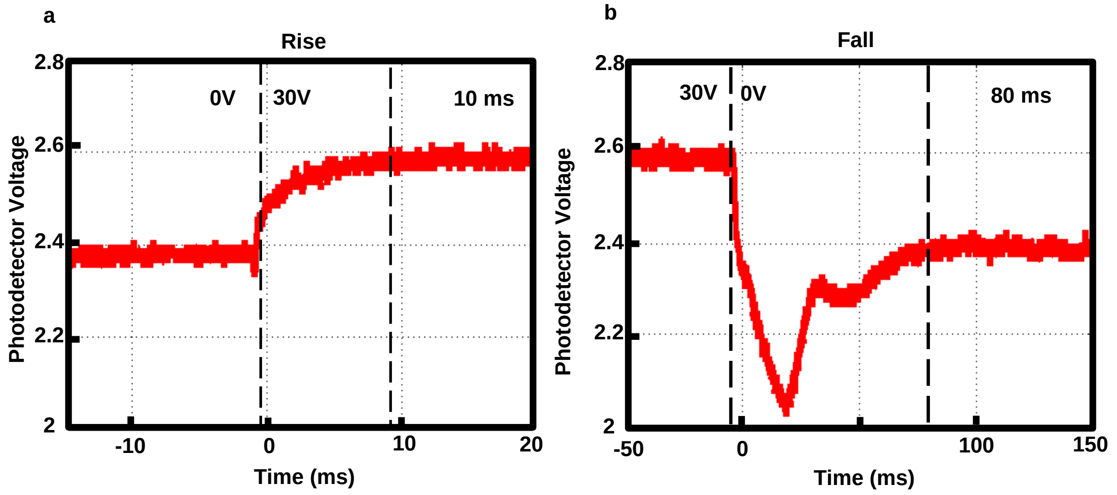

Supplementary Figure 9 | Response time measurements. Using a 633 nm He-Ne laser and photodiode, measurements of the response time can be made from 0 V μm^{-1} to 10 V μm^{-1} of the Afghan Girl in Fig. 5. (**a**) Shows the rise time (when voltage is applied) while (**b**) shows the fall time (when voltage is removed). Together, the measurements indicate a 90 ms cycling time.
